# 1,3-Bis(naphthalen-2-ylmeth­yl)-1*H*-anthra[1,2-*d*]imidazole-2,6,11(3*H*)-trione

**DOI:** 10.1107/S1600536811029102

**Published:** 2011-07-23

**Authors:** Zahra Afrakssou, Youssef Kandri Rodi, Frédéric Capet, El Mokhtar Essassi, Seik Weng Ng

**Affiliations:** a Laboratoire de Chimie Organique Appliquée, Faculté des Sciences et Techniques, Université Sidi Mohamed Ben Abdallah, Fés, Morocco; b Unité de Catalyse et de Chimie du Solide, Ecole Nationale Supérieure de Chimie de Lille, Lille, France; c Laboratoire de Chimie Organique Hétérocyclique, Pôle de Compétences Pharmacochimie, Université Mohammed V-Agdal, BP 1014 Avenue Ibn Batout, Rabat, Morocco; d Department of Chemistry, University of Malaya, 50603 Kuala Lumpur, Malaysia; e Chemistry Department, Faculty of Science, King Abdulaziz University, PO Box 80203 Jeddah, Saudi Arabia

## Abstract

The title compound, C_37_H_24_N_2_O_3_, is a 1*H*-anthra[2,1-*d*]imidazole-2,6,11(3*H*)-trione derivative having naphthyl­methyl substitutents attached to the imidazole N atoms. The anthraquinone part of the mol­ecule is somewhat folded along the the line connecting the carbonyl bonds. The dihedral angle between the two benzene rings is 7.8 (1)°. The two naphthyl systems of the substituents of the imidazole ring are positioned on the same side of the five-membered ring; these are approximately coplanar, the dihedral angle between the napthyl systems being 4.3 (2)°.

## Related literature

For the structure of 1,3-dibenzyl-1*H*-anthra[1,2-*d*]imidazole-2,6,11(3*H*)-trione, see: Afrakssou *et al.* (2011 ▶).
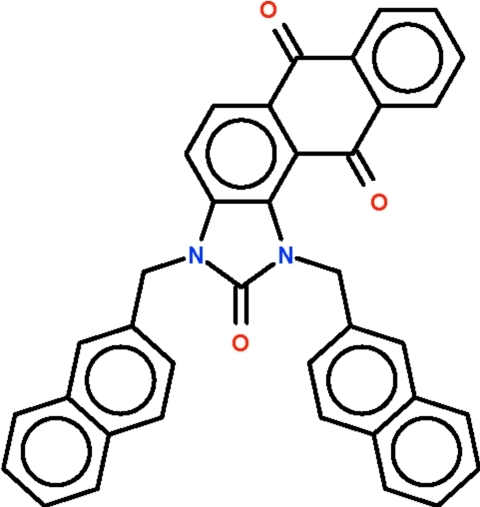


## Experimental

### 

#### Crystal data

C_37_H_24_N_2_O_3_
*M* *_r_* = 544.58
Orthorhombic, 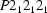

*a* = 8.0901 (1) Å
*b* = 12.8226 (2) Å
*c* = 26.1472 (4) Å
*V* = 2712.41 (7) Å^3^
*Z* = 4
Mo *K*α radiation
μ = 0.09 mm^−1^
*T* = 293 K
0.25 × 0.20 × 0.15 mm

#### Data collection

Bruker X8 APEXII diffractometer
34004 measured reflections
3781 independent reflections
3453 reflections with *I* > 2σ(*I*)
*R*_int_ = 0.026

#### Refinement

*R*[*F* ^2^ > 2σ(*F* ^2^)] = 0.044
*wR*(*F* ^2^) = 0.138
*S* = 1.10
3781 reflections
379 parameters
H-atom parameters constrained
Δρ_max_ = 0.33 e Å^−3^
Δρ_min_ = −0.15 e Å^−3^

### 

Data collection: *APEX2* (Bruker, 2008 ▶); cell refinement: *SAINT* (Bruker, 2008 ▶); data reduction: *SAINT*; program(s) used to solve structure: *SHELXS97* (Sheldrick, 2008 ▶); program(s) used to refine structure: *SHELXL97* (Sheldrick, 2008 ▶); molecular graphics: *X-SEED* (Barbour, 2001 ▶); software used to prepare material for publication: *publCIF* (Westrip, 2010 ▶).

## Supplementary Material

Crystal structure: contains datablock(s) global, I. DOI: 10.1107/S1600536811029102/bt6819sup1.cif

Structure factors: contains datablock(s) I. DOI: 10.1107/S1600536811029102/bt6819Isup2.hkl

Supplementary material file. DOI: 10.1107/S1600536811029102/bt6819Isup3.cml

Additional supplementary materials:  crystallographic information; 3D view; checkCIF report

## Figures and Tables

**Fig. 1.**
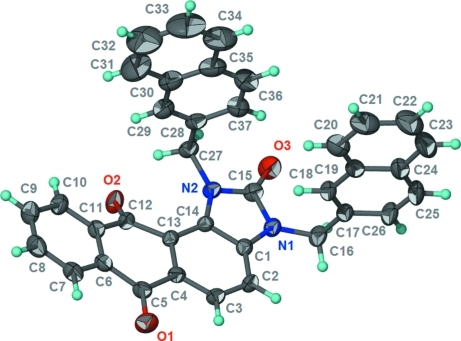
Anisotropic displacement ellipsoid plot (Barbour, 2001) of C37H24N2O3 at the 50% probability level; hydrogen atoms are drawn as arbitrary radius.

## supplementary crystallographic information

### Comment

The two nitrogen-bound H atoms of 1*H*-anthra [2,1-*d*]imidazole-2,6,11(3*H*)-trione can be replaced by a alkyl substitutent when the compound is reacted with an alkyl halide in a reaction catalyzed by tetra-*n*-butylammonium bromide; the di-benzyl substituted derivative is synthesized in such a synthesis in high yield. The study (Afrakssou *et al.*, 2011) is now extended to the title naphthylmethyl analog (Scheme I, Fig. 1). In the compound, C_37_H_24_N_2_O_3_, the anthraquinone part of the molecule is somewhat folded along the the line connecting the carbonyl bonds (dihedral angle between the two phenyl rings is 7.8 (1) °). The two naphthyl rings of the substituents of the imidazole ring are positioned on the same side of the five-membered ring; these are approximately coplanar (dihedral angle between napthyl rings is 4.3 (2) °).

### Experimental

To a solution of 1*H*-anthra [2,1-*d*]imidazole-2,6,11(3*H*)-trione (0.40 g, 1.51 mmol), potassium carbonate (0.83 g, 6.05 mmol) and tetra-*n*-butylammonium bromide (0.04 g, 0.15 mmol) in DMF (15 ml)) was added 2-(bromomethyl)naphthalene (0.83 g, 3.78 mmol). Stirring was continued at room temperature for 24 h. The mixture was filtered and the solvent removed. The residue was extracted with water. The organic compound was chromatographed on a column of silica gel with ethyl acetate-hexane (1/1) as eluent. Orange crystals were isolated when the solvent was allowed to evaporate.

### Refinement

Carbon-bound H-atoms were placed in calculated positions (C—H 0.93–0.97 Å) and were included in the refinement in the riding model approximation, with *U*(H) set to 1.2*U*(C). 2920 Friedel pairs were merged.

In the absence of heavy atoms, some 2920 Friedel pairs were merged.

### Crystal data

**Table:**

C_37_H_24_N_2_O_3_	*F*(000) = 1136
*M**_r_* = 544.58	*D*_x_ = 1.334 Mg m^−^^3^
Orthorhombic, *P*2_1_2_1_2_1_	Mo *K*α radiation, λ = 0.71073 Å
Hall symbol: P 2ac 2ab	Cell parameters from 9907 reflections
*a* = 8.0901 (1) Å	θ = 2.2–28.1°
*b* = 12.8226 (2) Å	µ = 0.09 mm^−^^1^
*c* = 26.1472 (4) Å	*T* = 293 K
*V* = 2712.41 (7) Å^3^	Prism, orange
*Z* = 4	0.25 × 0.20 × 0.15 mm

### Data collection

**Table:**

Bruker X8 APEXII diffractometer	3453 reflections with *I* > 2σ(*I*)
Radiation source: fine-focus sealed tube	*R*_int_ = 0.026
graphite	θ_max_ = 28.3°, θ_min_ = 1.6°
φ and ω scans	*h* = −10→10
34004 measured reflections	*k* = −16→17
3781 independent reflections	*l* = −34→33

### Refinement

**Table:**

Refinement on *F*^2^	Primary atom site location: structure-invariant direct methods
Least-squares matrix: full	Secondary atom site location: difference Fourier map
*R*[*F*^2^ > 2σ(*F*^2^)] = 0.044	Hydrogen site location: inferred from neighbouring sites
*wR*(*F*^2^) = 0.138	H-atom parameters constrained
*S* = 1.10	*w* = 1/[σ^2^(*F*_o_^2^) + (0.0817*P*)^2^ + 0.4504*P*] where *P* = (*F*_o_^2^ + 2*F*_c_^2^)/3
3781 reflections	(Δ/σ)_max_ = 0.001
379 parameters	Δρ_max_ = 0.33 e Å^−^^3^
0 restraints	Δρ_min_ = −0.15 e Å^−^^3^

### Fractional atomic coordinates and isotropic or equivalent isotropic displacement parameters (Å^2^)

**Table:**

	*x*	*y*	*z*	*U*_iso_*/*U*_eq_	
O1	0.8235 (3)	0.91407 (16)	0.22345 (10)	0.0663 (6)	
O2	0.2782 (2)	0.69294 (15)	0.20827 (8)	0.0520 (5)	
O3	0.5179 (3)	0.31578 (14)	0.17289 (9)	0.0622 (5)	
N1	0.7325 (3)	0.42761 (15)	0.19328 (7)	0.0405 (4)	
N2	0.4839 (2)	0.49636 (14)	0.17989 (7)	0.0376 (4)	
C1	0.7480 (3)	0.53404 (17)	0.19934 (8)	0.0356 (4)	
C2	0.8853 (3)	0.59225 (19)	0.21178 (9)	0.0415 (5)	
H2	0.9873	0.5609	0.2174	0.050*	
C3	0.8656 (3)	0.6996 (2)	0.21557 (9)	0.0414 (5)	
H3	0.9567	0.7412	0.2230	0.050*	
C4	0.7118 (3)	0.74609 (18)	0.20846 (8)	0.0361 (4)	
C5	0.7031 (3)	0.86234 (19)	0.21094 (9)	0.0427 (5)	
C6	0.5463 (3)	0.91261 (18)	0.19539 (8)	0.0402 (5)	
C7	0.5391 (4)	1.02130 (19)	0.18900 (9)	0.0490 (6)	
H7	0.6329	1.0619	0.1943	0.059*	
C8	0.3913 (4)	1.0677 (2)	0.17469 (10)	0.0555 (7)	
H8	0.3866	1.1395	0.1701	0.067*	
C9	0.2520 (4)	1.0085 (2)	0.16729 (11)	0.0583 (7)	
H9	0.1543	1.0405	0.1570	0.070*	
C10	0.2556 (3)	0.9011 (2)	0.17499 (10)	0.0495 (6)	
H10	0.1599	0.8617	0.1711	0.059*	
C11	0.4040 (3)	0.85298 (17)	0.18862 (8)	0.0392 (5)	
C12	0.4052 (3)	0.73869 (18)	0.19823 (8)	0.0372 (4)	
C13	0.5687 (3)	0.68596 (17)	0.19743 (8)	0.0328 (4)	
C14	0.5893 (3)	0.57835 (16)	0.19155 (7)	0.0327 (4)	
C15	0.5716 (3)	0.40268 (18)	0.18116 (9)	0.0428 (5)	
C16	0.8669 (3)	0.35269 (19)	0.18985 (10)	0.0481 (6)	
H16A	0.8266	0.2849	0.2005	0.058*	
H16B	0.9538	0.3732	0.2133	0.058*	
C17	0.9387 (3)	0.34343 (17)	0.13695 (10)	0.0429 (5)	
C18	0.9014 (3)	0.40959 (18)	0.09759 (10)	0.0451 (5)	
H18	0.8293	0.4648	0.1034	0.054*	
C19	0.9701 (3)	0.3961 (2)	0.04800 (10)	0.0484 (6)	
C20	0.9316 (4)	0.4642 (3)	0.00705 (12)	0.0637 (7)	
H20	0.8620	0.5207	0.0126	0.076*	
C21	0.9959 (5)	0.4474 (3)	−0.04074 (13)	0.0794 (10)	
H21	0.9703	0.4927	−0.0674	0.095*	
C22	1.1008 (5)	0.3617 (4)	−0.04943 (16)	0.0898 (13)	
H22	1.1429	0.3497	−0.0820	0.108*	
C23	1.1405 (5)	0.2970 (3)	−0.01072 (16)	0.0841 (12)	
H23	1.2102	0.2410	−0.0172	0.101*	
C24	1.0795 (3)	0.3116 (2)	0.03943 (13)	0.0601 (7)	
C25	1.1201 (4)	0.2469 (3)	0.08101 (14)	0.0707 (9)	
H25	1.1950	0.1928	0.0762	0.085*	
C26	1.0524 (4)	0.2618 (2)	0.12796 (13)	0.0621 (8)	
H26	1.0814	0.2175	0.1547	0.075*	
C27	0.3236 (3)	0.49777 (19)	0.15322 (9)	0.0425 (5)	
H27A	0.2505	0.5473	0.1698	0.051*	
H27B	0.2730	0.4293	0.1554	0.051*	
C28	0.3448 (3)	0.5277 (2)	0.09756 (10)	0.0464 (5)	
C29	0.2724 (4)	0.6134 (2)	0.07760 (12)	0.0582 (7)	
H29	0.2112	0.6569	0.0988	0.070*	
C30	0.2880 (4)	0.6389 (3)	0.02394 (12)	0.0637 (8)	
C31	0.2094 (8)	0.7273 (4)	0.00331 (19)	0.1075 (16)	
H31	0.1466	0.7711	0.0240	0.129*	
C32	0.2270 (9)	0.7476 (5)	−0.0475 (2)	0.124 (2)	
H32	0.1783	0.8069	−0.0614	0.149*	
C33	0.3171 (9)	0.6806 (5)	−0.07904 (17)	0.123 (2)	
H33	0.3223	0.6946	−0.1139	0.147*	
C34	0.3971 (7)	0.5962 (5)	−0.06056 (15)	0.1035 (15)	
H34	0.4607	0.5546	−0.0820	0.124*	
C35	0.3817 (5)	0.5718 (3)	−0.00662 (13)	0.0732 (9)	
C36	0.4562 (5)	0.4837 (3)	0.01427 (14)	0.0783 (10)	
H36	0.5183	0.4397	−0.0064	0.094*	
C37	0.4385 (4)	0.4619 (3)	0.06506 (12)	0.0638 (7)	
H37	0.4889	0.4028	0.0786	0.077*	

### Atomic displacement parameters (Å^2^)

**Table:**

	*U*^11^	*U*^22^	*U*^33^	*U*^12^	*U*^13^	*U*^23^
O1	0.0528 (11)	0.0477 (10)	0.0982 (16)	−0.0092 (9)	−0.0213 (11)	−0.0135 (11)
O2	0.0314 (8)	0.0470 (9)	0.0776 (13)	−0.0005 (7)	0.0068 (8)	0.0009 (9)
O3	0.0635 (12)	0.0356 (8)	0.0875 (15)	−0.0069 (9)	−0.0111 (11)	0.0065 (9)
N1	0.0402 (10)	0.0359 (9)	0.0454 (10)	0.0041 (8)	−0.0029 (8)	0.0030 (7)
N2	0.0343 (9)	0.0368 (9)	0.0418 (9)	−0.0045 (7)	−0.0033 (7)	0.0025 (7)
C1	0.0351 (10)	0.0383 (10)	0.0333 (9)	0.0020 (9)	0.0013 (8)	0.0009 (8)
C2	0.0287 (9)	0.0492 (12)	0.0465 (11)	0.0044 (9)	−0.0021 (9)	−0.0016 (10)
C3	0.0307 (10)	0.0475 (12)	0.0459 (11)	−0.0054 (9)	−0.0031 (9)	−0.0048 (10)
C4	0.0342 (10)	0.0396 (10)	0.0344 (10)	−0.0034 (9)	−0.0004 (8)	−0.0034 (8)
C5	0.0413 (12)	0.0409 (11)	0.0459 (12)	−0.0061 (10)	−0.0018 (10)	−0.0069 (9)
C6	0.0465 (12)	0.0382 (10)	0.0360 (10)	−0.0002 (10)	0.0000 (9)	−0.0061 (8)
C7	0.0627 (15)	0.0388 (11)	0.0456 (12)	−0.0025 (11)	0.0005 (11)	−0.0051 (9)
C8	0.0768 (19)	0.0363 (11)	0.0536 (14)	0.0082 (12)	−0.0006 (14)	−0.0017 (10)
C9	0.0631 (16)	0.0454 (13)	0.0666 (16)	0.0169 (13)	−0.0063 (14)	0.0005 (12)
C10	0.0449 (13)	0.0454 (12)	0.0582 (14)	0.0075 (11)	−0.0040 (11)	0.0011 (11)
C11	0.0415 (11)	0.0391 (10)	0.0372 (11)	0.0038 (9)	0.0014 (9)	−0.0009 (8)
C12	0.0323 (10)	0.0388 (10)	0.0406 (10)	0.0015 (8)	−0.0008 (8)	−0.0008 (8)
C13	0.0290 (9)	0.0382 (10)	0.0311 (9)	−0.0002 (8)	0.0012 (7)	0.0008 (7)
C14	0.0310 (9)	0.0377 (10)	0.0294 (8)	−0.0012 (8)	−0.0005 (7)	0.0007 (7)
C15	0.0460 (12)	0.0374 (10)	0.0449 (11)	−0.0030 (10)	−0.0025 (10)	0.0060 (9)
C16	0.0513 (14)	0.0390 (11)	0.0540 (14)	0.0114 (11)	−0.0060 (11)	0.0074 (10)
C17	0.0383 (11)	0.0326 (10)	0.0578 (13)	0.0037 (9)	−0.0077 (10)	−0.0053 (9)
C18	0.0432 (12)	0.0385 (11)	0.0535 (13)	0.0080 (10)	−0.0014 (10)	−0.0052 (9)
C19	0.0401 (12)	0.0501 (13)	0.0548 (13)	−0.0045 (11)	−0.0018 (10)	−0.0140 (11)
C20	0.0662 (18)	0.0668 (17)	0.0582 (16)	−0.0060 (16)	0.0029 (14)	−0.0079 (13)
C21	0.083 (2)	0.102 (3)	0.0535 (16)	−0.020 (2)	0.0029 (17)	−0.0042 (17)
C22	0.083 (3)	0.120 (3)	0.066 (2)	−0.014 (3)	0.018 (2)	−0.030 (2)
C23	0.064 (2)	0.096 (3)	0.092 (3)	0.002 (2)	0.016 (2)	−0.041 (2)
C24	0.0433 (13)	0.0601 (16)	0.0768 (18)	−0.0004 (13)	0.0029 (13)	−0.0247 (14)
C25	0.0575 (18)	0.0581 (17)	0.096 (2)	0.0236 (15)	−0.0032 (17)	−0.0215 (17)
C26	0.0557 (16)	0.0442 (13)	0.086 (2)	0.0194 (13)	−0.0112 (15)	−0.0067 (13)
C27	0.0316 (10)	0.0443 (11)	0.0517 (12)	−0.0095 (9)	−0.0044 (9)	−0.0003 (10)
C28	0.0399 (11)	0.0481 (13)	0.0512 (12)	−0.0100 (10)	−0.0136 (10)	0.0003 (10)
C29	0.0518 (14)	0.0605 (16)	0.0624 (16)	0.0023 (13)	−0.0105 (13)	−0.0016 (12)
C30	0.0605 (17)	0.0708 (18)	0.0599 (16)	−0.0016 (16)	−0.0144 (14)	0.0034 (14)
C31	0.120 (4)	0.115 (4)	0.088 (3)	0.024 (3)	−0.026 (3)	0.025 (3)
C32	0.152 (5)	0.144 (4)	0.076 (3)	0.003 (4)	−0.032 (3)	0.033 (3)
C33	0.151 (5)	0.164 (5)	0.053 (2)	−0.019 (5)	−0.033 (3)	0.028 (3)
C34	0.113 (4)	0.140 (4)	0.058 (2)	−0.012 (3)	−0.014 (2)	0.003 (2)
C35	0.068 (2)	0.092 (2)	0.0599 (17)	−0.0076 (19)	−0.0178 (15)	−0.0041 (17)
C36	0.081 (2)	0.093 (2)	0.0611 (17)	0.009 (2)	−0.0021 (17)	−0.0191 (18)
C37	0.0654 (18)	0.0679 (17)	0.0583 (15)	0.0064 (16)	−0.0097 (14)	−0.0098 (13)

### Geometric parameters (Å, °)

**Table:**

O1—C5	1.223 (3)	C18—H18	0.9300
O2—C12	1.212 (3)	C19—C20	1.416 (4)
O3—C15	1.215 (3)	C19—C24	1.417 (4)
N1—C15	1.377 (3)	C20—C21	1.370 (5)
N1—C1	1.380 (3)	C20—H20	0.9300
N1—C16	1.454 (3)	C21—C22	1.407 (6)
N2—C14	1.388 (3)	C21—H21	0.9300
N2—C15	1.395 (3)	C22—C23	1.347 (6)
N2—C27	1.473 (3)	C22—H22	0.9300
C1—C2	1.377 (3)	C23—C24	1.413 (5)
C1—C14	1.419 (3)	C23—H23	0.9300
C2—C3	1.390 (3)	C24—C25	1.406 (5)
C2—H2	0.9300	C25—C26	1.357 (5)
C3—C4	1.392 (3)	C25—H25	0.9300
C3—H3	0.9300	C26—H26	0.9300
C4—C13	1.420 (3)	C27—C28	1.515 (3)
C4—C5	1.494 (3)	C27—H27A	0.9700
C5—C6	1.480 (4)	C27—H27B	0.9700
C6—C11	1.394 (3)	C28—C29	1.350 (4)
C6—C7	1.405 (3)	C28—C37	1.418 (4)
C7—C8	1.387 (4)	C29—C30	1.446 (4)
C7—H7	0.9300	C29—H29	0.9300
C8—C9	1.373 (4)	C30—C35	1.398 (5)
C8—H8	0.9300	C30—C31	1.407 (6)
C9—C10	1.393 (4)	C31—C32	1.362 (7)
C9—H9	0.9300	C31—H31	0.9300
C10—C11	1.396 (3)	C32—C33	1.396 (9)
C10—H10	0.9300	C32—H32	0.9300
C11—C12	1.487 (3)	C33—C34	1.350 (8)
C12—C13	1.486 (3)	C33—H33	0.9300
C13—C14	1.398 (3)	C34—C35	1.450 (5)
C16—C17	1.505 (4)	C34—H34	0.9300
C16—H16A	0.9700	C35—C36	1.392 (6)
C16—H16B	0.9700	C36—C37	1.365 (5)
C17—C18	1.368 (3)	C36—H36	0.9300
C17—C26	1.413 (3)	C37—H37	0.9300
C18—C19	1.421 (4)		
			
C15—N1—C1	110.01 (19)	C19—C18—H18	119.2
C15—N1—C16	122.6 (2)	C20—C19—C18	122.0 (2)
C1—N1—C16	126.3 (2)	C20—C19—C24	119.3 (3)
C14—N2—C15	109.52 (18)	C18—C19—C24	118.8 (3)
C14—N2—C27	129.50 (19)	C21—C20—C19	120.6 (3)
C15—N2—C27	118.01 (19)	C21—C20—H20	119.7
C2—C1—N1	129.6 (2)	C19—C20—H20	119.7
C2—C1—C14	123.14 (19)	C20—C21—C22	119.9 (4)
N1—C1—C14	107.28 (19)	C20—C21—H21	120.1
C1—C2—C3	117.5 (2)	C22—C21—H21	120.1
C1—C2—H2	121.3	C23—C22—C21	120.2 (3)
C3—C2—H2	121.3	C23—C22—H22	119.9
C2—C3—C4	121.1 (2)	C21—C22—H22	119.9
C2—C3—H3	119.4	C22—C23—C24	122.2 (4)
C4—C3—H3	119.4	C22—C23—H23	118.9
C3—C4—C13	121.6 (2)	C24—C23—H23	118.9
C3—C4—C5	117.6 (2)	C25—C24—C23	123.9 (3)
C13—C4—C5	120.8 (2)	C25—C24—C19	118.3 (3)
O1—C5—C6	121.3 (2)	C23—C24—C19	117.8 (3)
O1—C5—C4	121.0 (2)	C26—C25—C24	121.5 (3)
C6—C5—C4	117.6 (2)	C26—C25—H25	119.3
C11—C6—C7	119.7 (2)	C24—C25—H25	119.3
C11—C6—C5	120.3 (2)	C25—C26—C17	121.1 (3)
C7—C6—C5	120.0 (2)	C25—C26—H26	119.4
C8—C7—C6	119.5 (3)	C17—C26—H26	119.4
C8—C7—H7	120.2	N2—C27—C28	111.01 (19)
C6—C7—H7	120.2	N2—C27—H27A	109.4
C9—C8—C7	120.6 (2)	C28—C27—H27A	109.4
C9—C8—H8	119.7	N2—C27—H27B	109.4
C7—C8—H8	119.7	C28—C27—H27B	109.4
C8—C9—C10	120.6 (3)	H27A—C27—H27B	108.0
C8—C9—H9	119.7	C29—C28—C37	119.0 (3)
C10—C9—H9	119.7	C29—C28—C27	121.9 (3)
C11—C10—C9	119.5 (3)	C37—C28—C27	119.1 (2)
C11—C10—H10	120.3	C28—C29—C30	121.5 (3)
C9—C10—H10	120.3	C28—C29—H29	119.3
C6—C11—C10	120.1 (2)	C30—C29—H29	119.3
C6—C11—C12	120.9 (2)	C35—C30—C31	121.5 (4)
C10—C11—C12	119.0 (2)	C35—C30—C29	117.5 (3)
O2—C12—C13	122.5 (2)	C31—C30—C29	121.0 (4)
O2—C12—C11	120.5 (2)	C32—C31—C30	118.7 (6)
C13—C12—C11	116.9 (2)	C32—C31—H31	120.6
C14—C13—C4	117.43 (19)	C30—C31—H31	120.6
C14—C13—C12	123.84 (19)	C31—C32—C33	120.9 (5)
C4—C13—C12	118.40 (18)	C31—C32—H32	119.6
N2—C14—C13	134.28 (19)	C33—C32—H32	119.6
N2—C14—C1	106.53 (17)	C34—C33—C32	122.2 (4)
C13—C14—C1	119.17 (19)	C34—C33—H33	118.9
O3—C15—N1	126.2 (2)	C32—C33—H33	118.9
O3—C15—N2	127.1 (2)	C33—C34—C35	118.7 (5)
N1—C15—N2	106.64 (19)	C33—C34—H34	120.7
N1—C16—C17	113.45 (19)	C35—C34—H34	120.7
N1—C16—H16A	108.9	C30—C35—C36	120.7 (3)
C17—C16—H16A	108.9	C30—C35—C34	118.0 (4)
N1—C16—H16B	108.9	C36—C35—C34	121.3 (4)
C17—C16—H16B	108.9	C37—C36—C35	120.2 (3)
H16A—C16—H16B	107.7	C37—C36—H36	119.9
C18—C17—C26	118.5 (3)	C35—C36—H36	119.9
C18—C17—C16	123.9 (2)	C36—C37—C28	121.1 (3)
C26—C17—C16	117.6 (2)	C36—C37—H37	119.4
C17—C18—C19	121.7 (2)	C28—C37—H37	119.4
C17—C18—H18	119.2		
			
C15—N1—C1—C2	179.5 (2)	C1—N1—C15—N2	−0.3 (3)
C16—N1—C1—C2	−11.8 (4)	C16—N1—C15—N2	−169.48 (19)
C15—N1—C1—C14	1.0 (3)	C14—N2—C15—O3	180.0 (3)
C16—N1—C1—C14	169.6 (2)	C27—N2—C15—O3	−17.6 (4)
N1—C1—C2—C3	−179.8 (2)	C14—N2—C15—N1	−0.5 (2)
C14—C1—C2—C3	−1.5 (3)	C27—N2—C15—N1	161.88 (19)
C1—C2—C3—C4	1.8 (3)	C15—N1—C16—C17	84.1 (3)
C2—C3—C4—C13	0.5 (3)	C1—N1—C16—C17	−83.2 (3)
C2—C3—C4—C5	−177.3 (2)	N1—C16—C17—C18	9.9 (4)
C3—C4—C5—O1	−6.7 (4)	N1—C16—C17—C26	−170.7 (2)
C13—C4—C5—O1	175.5 (2)	C26—C17—C18—C19	1.9 (4)
C3—C4—C5—C6	170.6 (2)	C16—C17—C18—C19	−178.6 (2)
C13—C4—C5—C6	−7.3 (3)	C17—C18—C19—C20	179.8 (3)
O1—C5—C6—C11	−170.5 (2)	C17—C18—C19—C24	0.2 (4)
C4—C5—C6—C11	12.3 (3)	C18—C19—C20—C21	−178.1 (3)
O1—C5—C6—C7	7.5 (4)	C24—C19—C20—C21	1.6 (5)
C4—C5—C6—C7	−169.7 (2)	C19—C20—C21—C22	0.2 (5)
C11—C6—C7—C8	−1.7 (4)	C20—C21—C22—C23	−1.1 (6)
C5—C6—C7—C8	−179.7 (2)	C21—C22—C23—C24	0.2 (6)
C6—C7—C8—C9	0.7 (4)	C22—C23—C24—C25	−178.9 (4)
C7—C8—C9—C10	1.3 (4)	C22—C23—C24—C19	1.6 (5)
C8—C9—C10—C11	−2.3 (4)	C20—C19—C24—C25	178.0 (3)
C7—C6—C11—C10	0.7 (3)	C18—C19—C24—C25	−2.3 (4)
C5—C6—C11—C10	178.7 (2)	C20—C19—C24—C23	−2.4 (4)
C7—C6—C11—C12	−176.2 (2)	C18—C19—C24—C23	177.3 (3)
C5—C6—C11—C12	1.8 (3)	C23—C24—C25—C26	−177.1 (3)
C9—C10—C11—C6	1.3 (4)	C19—C24—C25—C26	2.4 (5)
C9—C10—C11—C12	178.2 (2)	C24—C25—C26—C17	−0.3 (5)
C6—C11—C12—O2	156.5 (2)	C18—C17—C26—C25	−1.9 (4)
C10—C11—C12—O2	−20.5 (3)	C16—C17—C26—C25	178.6 (3)
C6—C11—C12—C13	−20.6 (3)	C14—N2—C27—C28	67.3 (3)
C10—C11—C12—C13	162.5 (2)	C15—N2—C27—C28	−91.0 (2)
C3—C4—C13—C14	−3.0 (3)	N2—C27—C28—C29	−119.1 (3)
C5—C4—C13—C14	174.77 (19)	N2—C27—C28—C37	63.1 (3)
C3—C4—C13—C12	170.7 (2)	C37—C28—C29—C30	0.3 (4)
C5—C4—C13—C12	−11.5 (3)	C27—C28—C29—C30	−177.5 (3)
O2—C12—C13—C14	21.4 (3)	C28—C29—C30—C35	−0.3 (5)
C11—C12—C13—C14	−161.59 (19)	C28—C29—C30—C31	178.7 (4)
O2—C12—C13—C4	−151.9 (2)	C35—C30—C31—C32	−0.6 (7)
C11—C12—C13—C4	25.1 (3)	C29—C30—C31—C32	−179.6 (5)
C15—N2—C14—C13	−177.5 (2)	C30—C31—C32—C33	1.9 (9)
C27—N2—C14—C13	22.8 (4)	C31—C32—C33—C34	−3.3 (10)
C15—N2—C14—C1	1.1 (2)	C32—C33—C34—C35	3.1 (9)
C27—N2—C14—C1	−158.6 (2)	C31—C30—C35—C36	−178.9 (4)
C4—C13—C14—N2	−178.4 (2)	C29—C30—C35—C36	0.1 (5)
C12—C13—C14—N2	8.3 (4)	C31—C30—C35—C34	0.5 (6)
C4—C13—C14—C1	3.2 (3)	C29—C30—C35—C34	179.5 (4)
C12—C13—C14—C1	−170.13 (19)	C33—C34—C35—C30	−1.7 (7)
C2—C1—C14—N2	−179.9 (2)	C33—C34—C35—C36	177.6 (5)
N1—C1—C14—N2	−1.3 (2)	C30—C35—C36—C37	0.0 (6)
C2—C1—C14—C13	−1.1 (3)	C34—C35—C36—C37	−179.3 (4)
N1—C1—C14—C13	177.57 (18)	C35—C36—C37—C28	0.0 (6)
C1—N1—C15—O3	179.2 (2)	C29—C28—C37—C36	−0.1 (5)
C16—N1—C15—O3	10.0 (4)	C27—C28—C37—C36	177.7 (3)
